# Inhibition of tumor autophagy: a strategy to improve anti-tumor immunity?

**DOI:** 10.1038/s41392-020-00429-8

**Published:** 2020-12-04

**Authors:** Andrew J. Freeman, Emily J. Lelliott, Jane Oliaro

**Affiliations:** 1 Cancer Immunology Program, Peter MacCallum Cancer Centre, Melbourne, VIC 3000 Australia; 2 Sir Peter MacCallum Department of Oncology, The University of Melbourne, Parkville, VIC 3010 Australia; 3 Department of Immunology and Pathology, Monash University, Melbourne, VIC 3004 Australia

**Keywords:** Tumour immunology, Cancer genetics

In a study recently published in *Nature*, Lawson et al. confirm several pathways previously implicated in tumor cell sensitivity to cytotoxic T lymphocytes (CTLs), and identify autophagy as an additional pathway involved in mediating tumor immune evasion.^1^

Autophagy is a regulated and conserved multistep process that enables cells to degrade and recycle intracellular components and is critical for cellular homeostasis. Autophagy has recently been associated with tumor immune evasion in pancreatic ductal adenocarcinoma,^2^ whereby this process selectively targeted the degradation of major histocompatibility complex I (MHC-I), limiting recognition, and anti-tumor responses by CTLs. Here, Lawson et al. identify an additional role for autophagy in tumor immune evasion, by mediating resistance to cytokine-mediated cell death.

**Fig. 1:**
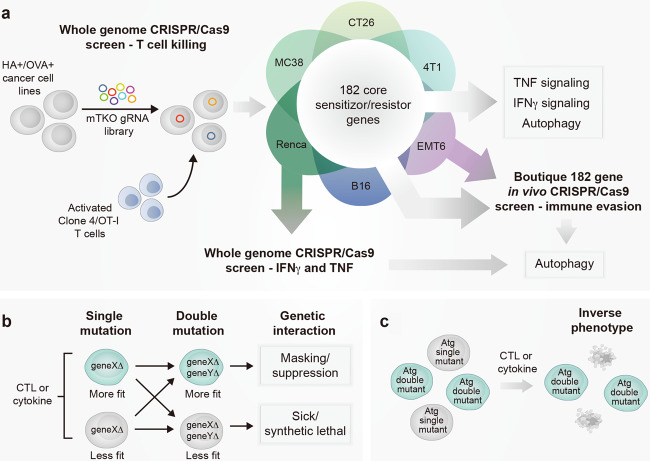
Autophagy genes regulate tumor immune evasion through complex genetic interactions. **a** Whole-genome CRISPR/Cas9 screen in multiple tumor cell lines identifies core genes mediating sensitivity or resistance to CTL killing. Further in vitro cytokine screening in RENCA cells and in vivo screening EMT6 cells identifies autophagy as a central mediator of tumor immune evasion. **b** Model of complex genetic interactions mediating cell fitness in response to CTL or cytokine pressure. **c** Single mutations and double mutations in autophagy genes result in an inverse phenotype in response to CTL or cytokine pressure, demonstrating an example of complex genetic interactions. HA—haemagglutinin model antigen target of Clone 4T cells; OVA—ovalbumin model antigen target of OT-I T cells; mTKO—mouse Toronto KnockOut library

Lawson et al. first utilized genome-scale clustered regularly interspaced short palindromic repeat (CRISPR)/CRISPR-associated protein 9 (Cas9) screening in multiple mouse cell lines with the aim of systematically identifying a fundamental group of genes which mediate sensitivity to antigen-specific CTL killing across a genetically diverse panel. The authors identified genes in the interferon-γ (IFN-γ) signaling and antigen presentation pathways as essential for antigen-specific CTL killing, consistent with previous studies using this technique.^3–5^ Genes involved in tumor necrosis factor (TNF)-mediated cell death in MC38 cells were also identified, supporting previous studies that implicate TNF as an important CTL anti-tumor effector mechanism.^5^ Genes that sensitized tumor cells to CTL killing when lost were also identified, and included genes involved in canonical TNF signaling and downstream nuclear factor κ-light-chain-enhancer of activated B cells (NF-κB) activation, as well as autophagy genes. Using these datasets, the authors identified a core group of 182 cancer-intrinsic CTL-evasion genes which were shared across the cell lines and included members of each of these pathways (Fig. 1a). Previous reports using pooled loss-of-function CRISPR/Cas9 screening with CTLs have focussed on one or two cell lines, arguably limiting the relevance of their findings to other tumor types. Lawson et al. overcome this through their analysis of several cancer cell lines and provide a comprehensive overview of the genes and pathways involved in CTL killing of diverse tumor cell types.

Given the strong dependence of IFN-γ signaling in their initial CRISPR/Cas9 screens, the authors next investigated the genes which protect against IFN-γ cytotoxicity. Again, utilizing CRISPR/Cas9 screening, the authors identified resistance and sensitizer genes in Renca cells after treatment with recombinant IFN-γ. Importantly, the authors identified various autophagy genes that sensitize the cells to IFN-γ when lost, previously identified in their core group of CTL-evasion genes. The authors then focus on the lipid-droplet-related gene, *Fitm2*, which scored as the top hit but has not previously been associated with tumor sensitivity to IFN-γ. Using a genetic interaction algorithm in combination with CRISPR/Cas9 screening, the authors delved deeper into the relationship between *Fitm2* loss and additional genetic perturbations which rendered cells either more or less sensitive to IFN-γ depending on their level of ‘fitness’ (Fig. 1b). Interestingly, the authors uncovered that loss of autophagy genes in the context of *Fitm2* loss resulted in resistance to IFN-γ, highlighting the importance of genetic interactions in tumor cell-CTL evasion. Surprisingly, this effect was absent in the presence of CTL, suggesting that autophagy loss may cause other effects in the presence of additional factors present during tumor-CTL interactions.

To investigate this further, and given the enrichment of TNF signaling genes amongst the sensitizer genes identified in their initial CRISPR/Cas9 screens, the authors next examined tumor regulators of TNF sensitivity. As expected, genome-wide screening of Renca cells with recombinant TNF identified genes regulating pro-survival NF-κB signaling as potent TNF-evasion genes. As with IFN-γ cytotoxicity, autophagy genes also regulated TNF sensitivity in this model, with tumor cells with a single mutation in autophagy genes exhibiting increased sensitivity to TNF cytotoxicity. In particular, the authors identified *Atg12* as a critical genetic determinant of autophagy-mediated immune evasion against TNF and CTLs. Genetic interaction analysis revealed that Renca cells lacking *Atg12* were more sensitive with additional genetic perturbations in downstream NF-κB signaling components, yet became less sensitive with perturbations in upstream components, highlighting a complex relationship between autophagy and NF-κB pathways during immune evasion in this model. Further validation experiments showed that *Atg12*-deficient Renca cells demonstrated normal NF-κB activation and transcriptional responses after exposure to TNF compared to control cells. However, they exhibited increased cytoplasmic expression of Sequestosome-1, an autophagosome cargo protein that binds and targets other proteins for autophagy. *Atg12*-deficient Renca cells also exhibited increased nuclear expression of nuclear factor erythroid 2-related factor 2, a transcription factor known to regulate autophagy. Although not empirically demonstrated, these findings suggest that *Atg12* may facilitate immune evasion by limiting the expression of these regulatory autophagy proteins, which may be part of an autophagy pathway or complex that possesses novel death-inducing activity in the presence of TNF. Again, using their genetic interaction algorithm, the authors unexpectedly identified that tumor cells with a double mutation in autophagy genes were paradoxically resistant to TNF or CTL killing (Fig. 1c). These findings highlight the complex nature of multiple genetic perturbations within the autophagy pathway and potential non-canonical functions of autophagy genes in immune evasion.

Finally, using CRISPR/Cas9 screening in EMT6 cells with a boutique library containing the previously identified 182 CTL-evasion genes, the authors validated their earlier findings in an in vivo setting (Fig. 1a). A gene representation comparison of late library-transduced tumors in immune-competent mice versus immune-compromised mice showed a significant depletion of numerous autophagy genes, confirming their substantial protective effects against endogenous immune cell killing amongst known evasion genes. Here the selection pressure was the endogenous immune response, and not specific to CTLs, suggesting that autophagy protects tumors from various immune cells within the tumor microenvironment, most likely those with the ability to secrete TNF and IFN-γ. The presence of antigen presentation genes among their sensitizer genes suggests a strong contribution of natural killer cells in this effect.

In summary, Lawson et al. provide a comprehensive analysis of the genes and pathways that mediate tumor immune evasion across various tumor models, and further highlight inhibition of tumor autophagy as a potential strategy to augment CTL killing and immunotherapy responses. Importantly, these effects were mediated through CTL-secreted TNF and IFN-γ and independent of MHC-I. Their findings additionally bring to attention the concept of target specificity and the effects of complex genetic interactions that may complicate targeting of single genes during immunotherapy. While this may have important implications in tumor responses to immunotherapy, an essential extension of this work would be to examine if similar interactions are observed at the protein level with pharmacological agents that target autophagy. If so, this could potentially limit the utility of targeting autophagy to augment CTL-driven anti-tumor immunity.
